# Supplementary material for: Content-rich biological network constructed by mining PubMed abstracts
Source: BMC Bioinformatics. 2004 Oct 8;5:147. doi: 10.1186/1471-2105-5-147 (PMC528731; doi:10.1186/1471-2105-5-147)
Supplement: Additional File 2 — The original results of the above study (non-essential files are deleted to keep the file size under the limit set by BMC bioinformatics). [file 1471-2105-5-147-S2.bz2 › chilibotAdditionalFile2/dip10/313ID10606357E1370/html/S100A8_S100A9.html]

 


 **S100A8** and **S100A9** 
  
Found 177 abstracts in PubMed, retrieved 10.  
 

 What does Google say? 
 PDF only 
| .edu only 

---

**Interactive relationship** (e.g. stimulation, inhibition, etc)

**Stimulatory relationship**- An abundant 32kDa protein capable of stimulating IL 8 production was isolated from the secretion and identified as neutrophil cytoplasmic protein MRP 14  [ **S100A9** ] , which is the heavy polypeptide chain in the MRP 8  [ **S100A8** ]  14 heterodimer.  Ref: 12748056 Am J Respir Cell Mol Biol< MedlineTA>Am J Respir Cell Mol Biol,
**Neutral relationship**- Moreover, MRP8  [ **S100A8** ]  MRP14  [ **S100A9** ]  initiated oxidase activation on its own, through a calcium dependent specific interaction with cytochrome b558 as shown by Atomic Force Microscopy and a structure function relationship investigation.  Ref: 12719414 J Biol Chem< MedlineTA>J Biol Chem,
- There was also an upregulation of  **S100A8**  A9 or A12 protein, as detected by the antibody MAC387  [ **S100A9** ] , in normal keratinocytes adjacent to both atypical keratinocytes and melanocytes in PAK and LM, respectively.  Ref: 12652189 Am J Dermatopathol, 2003

**Non-interactive relationship** (e.g. studied together, co-existance, homology, etc.)

- MRP8  [ **S100A8** ]  and MRP14  [ **S100A9** ]  were co isolated from neutrophils on an anti p47 phox matrix with oxidase cytosolic factors and identified by mass spectrometry.  Ref: 12719414 J Biol Chem< MedlineTA>J Biol Chem, 2003
- ... S100A7,  **S100A8** , and  **S100A9**  are markedly overexpressed.  Ref: 12704215 J Histochem Cytochem, 2003
- MRP8  [ **S100A8** ]  MRP14  [ **S100A9** ]  levels in serum correlated with systemic parameters of disease activity erythrocyte sedimentation rate ESR r = 0.55, P = 0.005.  Ref: 12794836 Arthritis Rheum, 2003
- Calprotectin  **S100A8**   **S100A9** , an Inflammatory Protein Complex from Neutrophils with a Broad Apoptosis Inducing Activity.  Ref: 12808281 Biol Pharm Bull, 2003
- In a semi recombinant cell free assay, recombinant MRP8  [ **S100A8** ]  MRP14  [ **S100A9** ]  increased the affinity of p67 phox for cytochrome b558 synergistically with p47 phox.  Ref: 12719414 J Biol Chem< MedlineTA>J Biol Chem, 2003
- Expression of MRP8  [ **S100A8** ] , MRP14  [ **S100A9** ] , and MRP8  [ **S100A8** ]  MRP14  [ **S100A9** ]  in synovium was determined by immunohistochemistry.  Ref: 12794836 Arthritis Rheum, 2003
- Our present studies compare the level of expression, and cellular localization of S100A7,  **S100A8** ,  **S100A9** , S100A10, and S100A11 in normal and psoriatic epidermis.  Ref: 12704215 J Histochem Cytochem, 2003
- A novel inflammatory syndrome defined by extraordinarily high expression of  **S100A8**  and  **S100A9**  confirmed recent observations in vitro demonstrating a role of these proteins during recruitment of leukocytes.  Ref: 12697438 Trends Immunol, 2003
- Myeloid related proteins MRP8  [ **S100A8** ]  and MRP14  [ **S100A9** ]  of the S100 family are EF hand calcium binding proteins.  Ref: 12719414 J Biol Chem< MedlineTA>J Biol Chem, 2003
- Changing the conformation state of cytochrome b558 initiates NADPH oxidase activation MRP8  [ **S100A8** ]  MRP14  [ **S100A9** ]  regulation.  Ref: 12719414 J Biol Chem< MedlineTA>J Biol Chem, 2003
- **S100A8**  and  **S100A9**  are absent or are expressed at minimal levels in normal epidermis.  Ref: 12704215 J Histochem Cytochem, 2003
- These studies also indicate, as reported previously, that S100A7,  **S100A8** , and  **S100A9**  expression is markedly altered in psoriasis, suggesting a role for these proteins in disease pathogenesis.  Ref: 12704215 J Histochem Cytochem, 2003
- This was confirmed ex vivo by co transfection of EBV transformed B lymphocytes with genes encoding MRP8  [ **S100A8** ]  and MRP14  [ **S100A9** ] .  Ref: 12719414 J Biol Chem< MedlineTA>J Biol Chem, 2003
- **S100A8**  and  **S100A9**  are strongly expressed in the basal and spinous layers in psoriasis involved tissue.  Ref: 12704215 J Histochem Cytochem, 2003
- MRP8  [ **S100A8** ]  MRP14  [ **S100A9** ]  purified from neutrophils, enhanced oxidase turnover of B cells in vitro, suggesting that MRP8  [ **S100A8** ]  MRP14  [ **S100A9** ]  is involved in the activation process.  Ref: 12719414 J Biol Chem< MedlineTA>J Biol Chem, 2003
- **S100A8**  and  **S100A9**  did not form these larger complexes.  Ref: 12704215 J Histochem Cytochem, 2003
- Moreover, MRP8  [ **S100A8** ]  MRP14  [ **S100A9** ]  dimer behaves as a positive mediator of phagocyte NADPH oxidase regulation.  Ref: 12719414 J Biol Chem< MedlineTA>J Biol Chem, 2003
- In contrast, MRP8  [ **S100A8** ] , MRP14  [ **S100A9** ] , and MRP8  [ **S100A8** ]  MRP14  [ **S100A9** ]  expression in the SLL of PsA patients is increased, particularly in perivascular regions, compared with that in RA and SpA patients, suggesting a central role of MRP proteins in transendothelial migration of leukocytes in PsA.  Ref: 12794836 Arthritis Rheum, 2003
- Together with two other calgranulins,  **S100A8**  and  **S100A9** , it is mostly expressed in human granulocytes, although there is increasing evidence of expression in keratinocytes and psoriatic lesions.  Ref: 12645006 Microsc Res Tech, 2003
- In addition, it was verified by immunohistochemical analysis that the expressions of Calgranulin A  [ **S100A8** ] , Calgranulin B  [ **S100A9** ] , and Cathepsin D were mainly located in cholesteatoma epithelium.  Ref: 12792315 Laryngoscope, 2003
- Concentrations of MRP8  [ **S100A8** ]  MRP14  [ **S100A9** ]  in serum and SF were measured by enzyme linked immunosorbent assay.  Ref: 12794836 Arthritis Rheum, 2003
- OBJECTIVE To analyze S 100 protein expression, in the form of myeloid related protein 8 MRP8  [ **S100A8** ] , MRP14  [ **S100A9** ] , and the heterodimer MRP8  [ **S100A8** ]  MRP14  [ **S100A9** ] , in psoriatic arthritis PsA patients compared with rheumatoid arthritis RA and spondylarthropathy SpA patients, and to determine the effect of methotrexate MTX on the MRP antigen expression in PsA patients.  Ref: 12794836 Arthritis Rheum, 2003
- These included genes involved in cell proliferation and differentiation eg, calgranulin A  [ **S100A8** ] , calgranulin B  [ **S100A9** ] , psoriasin, thymosin beta 10 and cell invasion eg, cathepsin C, cathepsin D, cathepsin H.  Ref: 12792315 Laryngoscope, 2003
- Analyses by means of reverse transcription polymerase chain reaction showed enhanced expression of several genes including calgranulin A  [ **S100A8** ] , calgranulin B  [ **S100A9** ] , psoriasin, thymosin beta 10, cathepsin C, cathepsin D, and cathepsin H in cholesteatoma, supporting the findings from the gene array.  Ref: 12792315 Laryngoscope, 2003
- RESULTS MRP8  [ **S100A8** ] , MRP14  [ **S100A9** ] , and MRP8  [ **S100A8** ]  MRP14  [ **S100A9** ]  levels were increased in serum, SF, and synovium from PsA, RA, and SpA patients.  Ref: 12794836 Arthritis Rheum, 2003
- In all 3 groups, paired samples of serum and SF showed significantly higher MRP8  [ **S100A8** ]  MRP14  [ **S100A9** ]  levels in SF mean SD 1 10 1 99 ng ml median 1 00 than in serum 908 679 ng ml median 695 P = 0.0001.  Ref: 12794836 Arthritis Rheum, 2003
- Together with  **S100A8**  and  **S100A9** , it belongs to the calgranulin subfamily, i.e. it is mainly expressed in granulocytes, although there is an increasing body of evidence of expression in keratinocytes and psoriatic lesions.  Ref: 12777802 Acta Crystallogr D Biol Crystallogr, 2003
- MRP8  [ **S100A8** ]  and MRP14  [ **S100A9** ]  are absent from Epstein Barr virus immortalized B lymphocytes and coincidentally, these cells display weak oxidase activity compared with neutrophils.  Ref: 12719414 J Biol Chem< MedlineTA>J Biol Chem, 2003
